# Correction to “Trends in Primary Biliary Cholangitis: Prospective Cohort Study From the European Reference Network Registry (R‐LIVER)”

**DOI:** 10.1002/ueg2.70201

**Published:** 2026-03-20

A. Gerussi, E. Nofit, D. P. Bernasconi, et al. 2025. “Trends in Primary Biliary Cholangitis: Prospective Cohort Study From the European Reference Network Registry (R‐LIVER),” *United European Gastroenterology Journal*: 13. no. 10, 1955–1963. https://doi.org/10.1002/ueg2.70134.

Piotr Milkiewicz’s name and affiliation have been corrected to include Translational Medicine Group, Pomeranian Medical University, Szczecin, Poland.

